# α_1_ adrenoceptor activation by norepinephrine inhibits LPS-induced cardiomyocyte TNF-α production *via* modulating ERK1/2 and NF-κB pathway

**DOI:** 10.1111/jcmm.12184

**Published:** 2013-12-05

**Authors:** Xiaohui Yu, Baoyin Jia, Faqiang Wang, Xiuxiu Lv, Xuemei Peng, Yiyang Wang, Hongmei Li, Yanping Wang, Daxiang Lu, Huadong Wang

**Affiliations:** a Department of Pathophysiology, Key Laboratory of State Administration of Traditional Chinese Medicine of the People's Republic of China, School of Medicine, Jinan UniversityGuangzhou, Guangdong, China; b Department of Anesthesiology, The First Affiliated Hospital, Jinan UniversityGuangzhou, Guangdong, China

**Keywords:** α_1_-adrenoceptor, Lipopolysaccharide, Tumour necrosis factor-α, cardiomyocytes

## Abstract

Cardiomyocyte tumour necrosis factor α (TNF-α) production contributes to myocardial depression during sepsis. This study was designed to observe the effect of norepinephrine (NE) on lipopolysaccharide (LPS)-induced cardiomyocyte TNF-α expression and to further investigate the underlying mechanisms in neonatal rat cardiomyocytes and endotoxaemic mice. In cultured neonatal rat cardiomyocytes, NE inhibited LPS-induced TNF-α production in a dose-dependent manner. α_1_-adrenoceptor (AR) antagonist (prazosin), but neither β_1_-nor β_2_-AR antagonist, abrogated the inhibitory effect of NE on LPS-stimulated TNF-α production. Furthermore, phenylephrine (PE), an α_1_-AR agonist, also suppressed LPS-induced TNF-α production. NE inhibited p38 phosphorylation and NF-κB activation, but enhanced extracellular signal-regulated kinase 1/2 (ERK1/2) phosphorylation and c-Fos expression in LPS-treated cardiomyocytes, all of which were reversed by prazosin pre-treatment. To determine whether ERK1/2 regulates c-Fos expression, p38 phosphorylation, NF-κB activation and TNF-α production, cardiomyocytes were also treated with U0126, a selective ERK1/2 inhibitor. Treatment with U0126 reversed the effects of NE on c-Fos expression, p38 mitogen-activated protein kinase (MAPK) phosphorylation and TNF-α production, but not NF-κB activation in LPS-challenged cardiomyocytes. In addition, pre-treatment with SB202190, a p38 MAPK inhibitor, partly inhibited LPS-induced TNF-α production in cardiomyocytes. In endotoxaemic mice, PE promoted myocardial ERK1/2 phosphorylation and c-Fos expression, inhibited p38 phosphorylation and IκBα degradation, reduced myocardial TNF-α production and prevented LPS-provoked cardiac dysfunction. Altogether, these findings indicate that activation of α_1_-AR by NE suppresses LPS-induced cardiomyocyte TNF-α expression and improves cardiac dysfunction during endotoxaemia *via* promoting myocardial ERK phosphorylation and suppressing NF-κB activation.

## Introduction

Myocardial depression has been identified as a major contributor to mortality in septic patients 1. It is well-established that tumour necrosis factor-α (TNF-α) is an important inducer of myocardial depression during sepsis 2. Administration of TNF-α directly depresses myocardial contractile function in animals and human cardiomyocytes 3,4, and anti-TNF-α therapy preserves myocardial function in endotoxaemic animals and septic patients 5,6. During sepsis, lipopolysaccharide (LPS) is recognized as the important pathogen-associated molecular pattern responsible for stimulating TNF-α production 3,7. Lipopolysaccharide stimulates Toll-like receptor 4 (TLR4) on immune cells and cardiomyocytes, activates mitogen-activated protein kinase (MAPK) kinases and inhibitors of κB (IκB) kinases, leading to the phosphorylation of p38 MAPK, extracellular signal-regulated kinase 1/2 (ERK1/2), c-Jun N-terminal kinases (JNK) and IκB, as well as subsequent activation of nuclear factor-κB (NF-κB), which induce and regulate TNF-α expression 2,8,9. Although it was reported that TNF-α produced by infiltrating and resident macrophages was responsible for LPS-induced myocardial dysfunction 10, recent studies have demonstrated that TLR4-mediated TNF-α production in cardiomyocytes plays a key role in LPS-induced cardiac depression 11,12. Therefore, insights into the regulatory mechanisms of cardiomyocyte TNF-α expression may provide a therapeutic modality for cardiac dysfunction during sepsis.

A growing body of evidence suggests that the nervous system plays a critical role in precise modulation of exaggerated innate immune response in sepsis *via* different hormonal and neuronal routes, such as sympathetic nervous pathway 13. Clinical studies have shown a significant increase in plasma concentrations of catecholamines, especially norepinephrine (NE) in septic patients 14,15. Experimental observations also confirmed that plasma NE level markedly increased in septic rats 16. Elevated NE regulates inflammatory cytokine expression during sepsis *via* a group of adrenergic receptor subtypes expressed on innate immune cells 13. For example, NE potentiated LPS-induced TNF-α release in macrophages *via* binding to α_2_-AR and increasing MAPK phosphorylation 17,18. In contrast, epinephrine and high doses of NE activated β-AR and down-regulated LPS-induced TNF-α production from macrophages 13.

As mentioned above, LPS also induces TNF-α expression in cardiomyocytes 2. Moreover, it is well recognized that α_1_-AR and β-AR exist in cardiomyocytes and NE is often used for the treatment of septic shock as the first choice of vasopressors 19,20. However, it remains unclear whether NE affects LPS-induced TNF-α expression in cardiomyocytes. Therefore, this study was designed to examine the effect of NE on LPS-induced cardiomyocyte TNF-α expression and the underlying molecular mechanisms. Our data demonstrated that NE inhibited LPS-induced cardiomyocyte TNF-α expression through regulating ERK phosphorylation and NF-κB activation in an α_1_-AR-dependent manner.

## Materials and methods

### Animals

The neonatal Sprague–Dawley rats (2–3 days old) and Male BALB/c mice (8–10 weeks old) were purchased from the medical laboratory animal centre of Guangdong province (Guangzhou, China). The experimental protocols were approved by the Experimental Animal Care and Use Committee of School of Medicine, Jinan University, which conform to the Guide for the Care and Use of Laboratory Animals published by the US National Institutes of Health (NIH Publication No 85-23, revised 1996). All surgery was performed under anaesthesia, and every effort was made to minimize suffering.

### Neonatal rat cardiomyocyte culture and treatment

Cardiomyocytes were prepared from the hearts of 2-to 3-day-old neonatal Sprague–Dawley rats as described previously 21. After 48 hrs of culture, cardiomyocytes (1 × 10^5^ cells/ml) were treated with vehicle or NE (Sigma-Aldrich, St. Louis, MO, USA) at concentrations of 2 nM–2 μM or phenylephrine (PE, a selective α_1_-AR agonist) at doses of 0.2–20 μM for 10 min., and followed by normal saline or LPS (1 μg/ml; Escherichia coli, 055:B5, Sigma-Aldrich) treatment. In the separate experiment, cardiomyocytes were pre-incubated with prazosin (a selective α_1_-AR antagonist), atenolol (a selective β_1_-AR antagonist), ICI-118,551(a selective β_2_-AR antagonist), U0126 (a highly selective inhibitor of ERK1/2) or SB 202190 (a selective inhibitor of p38 MAPK; Sigma-Aldrich) for 30 min. before treatment with NE or/and LPS respectively. Moreover, the cell viability was measured using the Cell Counting kit-8 (Dojindo Molecular Technologies Inc., Kumamoto, Japan).

### ELISA

The levels of TNF-α in the supernatants and plasma were determined using TNF-α ELISA kits (R&D Systems, Minneapolis, MN, USA) according to the manufacturer's instructions.

### Analysis of TNF-α mRNA by real-time PCR

Total RNA was isolated from cardiomyocytes using Trizol reagent and was reverse transcribed using a PrimeScript® RT reagent kit. Real-time PCR were performed with the SYBR® PrimeScript™ RT-PCR Kit II (TaKaRa, Kyoto, Japan), and the reactions were carried out in a LC480 real-time PCR system (Roche, Basel, Switzerland). The nucleotide sequences of primers used were as follows: TNF-α (forward 5′-ATACACTGGCCCGAGGCAAC-3′ and reverse 5′-CCACATCTCGGATCATGCTTTC-3′) and GAPDH (forward 5′-GGCACAGTCAAGGCTGAGAATG-3′ and reverse 5′-ATGGTGGTGAAGACGCCAGTA-3′). The TNF-α gene signal was normalized to GAPDH.

### Immunofluorescence examination of NF-κB nuclear translocation

After treatment, cardiomyocytes were fixed in paraformaldehyde (4%) for 30 min. at room temperature, and then permeabilized with Triton X-100 (0.5% in PBS) at 4°C for 5 min. After blocking with 5% normal goat serum, cardiomyocytes were incubated with rabit-anti-NF-κB p65 (1:50) primary antibody and mouse-anti-cardiac troponin I (1:50) antibody (Cell Signalling Technology Inc., Danvers, MA, USA) at 4°C overnight. After washing in PBS, cardiomyocytes were incubated with FITC-conjugated-anti-rabbit IgG and Alexa-fluo-conjugated antimouse secondary antibody (Abcam plc, Cambridge, UK) at 37°C for 30 min. Subsequently, 4′,6-diamidino-2-phenylindole was added for another 10 min. in the dark. Then, cells were observed by a laser-scanning confocal microscope (LSM510META; Zeiss, Oberkochen, Germany).

### Experimental design *in vivo*

Male BALB/c mice were allowed to acclimate for at least 3 days before the experimentation in the standard laboratory (24 ± 2°C and 12 hrs light/dark cycle) with free access to mouse chow and water. The mice were randomly divided into four groups: The control group, LPS group, PE+LPS group and PE group. Animals received subcutaneous injection of normal saline or PE 30 min. before and 2 hrs after saline or 20 mg/kg LPS administration. At 12 hrs after LPS administration, the echocardiography examination was performed. In another experiment, the mouse hearts and plasma were harvested at 2.5 hrs after LPS treatment under anaesthesia with pentobarbital sodium (100 mg/kg, i.p.) for western blotting and ELISA analysis.

### Echocardiography examination

The M-mode and Doppler transthoracic echocardiography examinations were performed with a VisualSonics Vevo770™ High-Resolution In Vivo Imagine System (VisualSonics Inc, Toronto, ON, Canada) with a 30-MHz centre frequency RMV 707 scan head (VisualSonics Inc) at 12 hrs after LPS or normal saline injection as previously described 22. Parameters including LV ejection fraction (EF), fractional shortening (FS), stroke volume (SV) and cardiac output (CO) were calculated by the software of Vevo770™ imaging system. Ascending aortic flow velocity was detected using the continuous Doppler wave mode for calculation of SV. The echocardiography measurements were interpreted by the investigator blinded to treatment, and the data were averaged from at least three consecutive cardiac cycles.

### Western blot analysis

Neonatal rat cardiomyocytes or the mouse heart homogenates were harvested in RIPA lysis buffer (Bioteke Co, Beijing, China) containing 1 mM phenylmethylsulfonyl fluoride and then centrifuged at 12,000 × *g* for 15 min. at 4°C. Cytosolic and nuclear proteins for NF-κB detection were prepared using NE-PER® nuclear and cytoplasmic extraction reagents (Thermo scientific, Rockford, IL, USA). Whole cell or tissue lysates were used for analysis unless otherwise specified. Equal amounts of protein were separated by SDS-PAGE and transferred onto a nitrocellulose membrane. The membranes were incubated with appropriate primary antibodies against c-Fos, NF-κB, p38, phospho-p38 (Thr180/Tyr182), ERK1/2, phospho-ERK1/2 (Thr202/Tyr204), JNK1/2, phospho-JNK1/2 (Thr183/Tyr185), IκBα, phospho-IκBα (Ser32), lamin B1, GAPDH (Cell Signalling Technology Inc.) or TNF-α (R&D System), respectively, as previously reported 21, followed by detection with an enhanced chemiluminescence advance western blot detection kit (Millipore, Billerica, MA, USA) after incubated with a horseradish peroxidase–conjugated goat anti-rabbit or mouse IgG secondary antibody. The bands were quantified by optical density ratio using GAPDH as a control. In the case of nuclear NF-κB, lamin B1 was employed as the loading control.

### Statistical analysis

Data were expressed as mean ± SEM and analysed using statistical software SPSS 13.0 (SPSS Inc., Chicago, IL, USA). The significance of the differences among groups was determined by one-way anova with the post hoc Tukey's honest significant difference test. Statistical significance was accepted at *P* < 0.05.

## Results

### NE inhibits LPS-induced TNF-α release from neonatal rat cardiomyocytes

**Fig 1:**
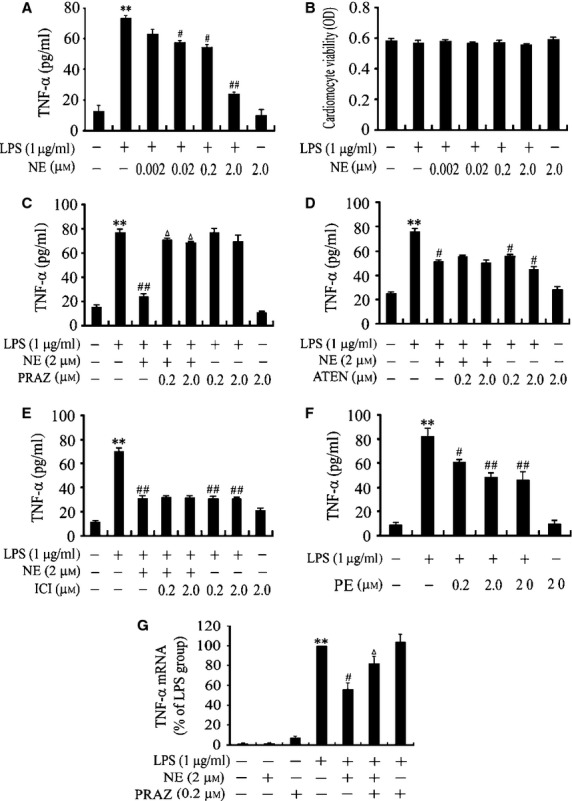
Norepinephrine (NE) inhibits lipopolysaccharide (LPS)-induced tumour necrosis factor-α production *via* activating α_1_ adrenoceptor in neonatal rat cardiomyocytes. (A, B and F) Cardiomyocytes were treated with NE, phenylephrine (PE) or vehicle for 10 min. and then with LPS or normal saline for 6 hrs. (C–E and G) After pre-treatment with prazosin (PRAZ), atenolol (ATEN) or ICI-118,551 (ICI) for 30 min., cardiomyocytes were stimulated with NE for 10 min. and with LPS for another 6 hrs (C–E) or 1.5 hrs (G). Data are mean ± SEM from four independent experiments. **P < 0.01 *versus* control, ^#^*P* < 0.05, ^#*#*^*P* < 0.01 *versus* LPS group, ^Δ^*P* < 0.05 *versus* LPS+NE group.

As shown in Figure 1A, LPS at 1 μg/ml induced a significant increase in TNF-α release from cardiomyocytes by 581% compared with control group (*P* < 0.01). Treatment with NE (20 nM–2 μM) caused a dose-dependent inhibition (by 26.8%, 28.3%, 67.4%) of TNF-α production in cardiomyocytes stimulated with LPS for 6 hrs, but NE alone did not affect TNF-α production. In addition, the indicated drugs did not affect viability of cardiomyocytes (Fig. 1B).

### Contribution of α_1_-AR activation to the inhibition of TNF-α production by NE in LPS-challenged cardiomyocytes

We further investigated the role of α_1_-, β_1_-and β_2_-AR in the inhibition of TNF-α expression by NE in LPS-challenged cardiomyocytes. Cardiomyocytes were pre-treated with prazosin, atenolol, ICI 118,551 or vehicle for 30 min. following incubation with NE at 2 μM or vehicle for 10 min. Then, the cardiomyocytes were further stimulated with LPS for 1.5 or 6 hrs; the TNF-α mRNA expression in cardiomyocytes and TNF-α level in the medium were examined. As described in Figure 1C–E and G, NE significantly inhibited LPS-induced TNF-α production and mRNA expression by 35% in cardiomyocytes, which were reversed by pre-treatment with prazosin. In contrast, neither atenolol nor ICI 118,551 abrogated the inhibitory effect of NE on LPS-stimulated TNF-α production. However, both atenolol and ICI 118,551 suppressed TNF-α production in LPS-treated cardiomyocytes. Furthermore, pre-treatment with PE (an α_1_-AR agonist, 0.2 μM–20 μM) for 10 min. significantly decreased LPS-induced TNF-α production by 21%, 41% and 44% in cardiomyocytes respectively (Fig. 1F). In addition, prazosin, atenolol, ICI 118,551 or PE alone did not affect TNF-α production in cardiomyocytes; the indicated treatment had no significant effects on the viability of cardiomyocytes (data not shown). These findings indicate that α_1_-AR is necessary for the inhibitory effect of NE on TNF-α production in LPS-treated cardiomyocytes.

### Norepinephrine suppresses p38 MAPK and NF-κB activation, enhances ERK1/2 phosphorylation and c-Fos expression *via* α_1_-AR in LPS-challenged cardiomyocytes

**Fig 2:**
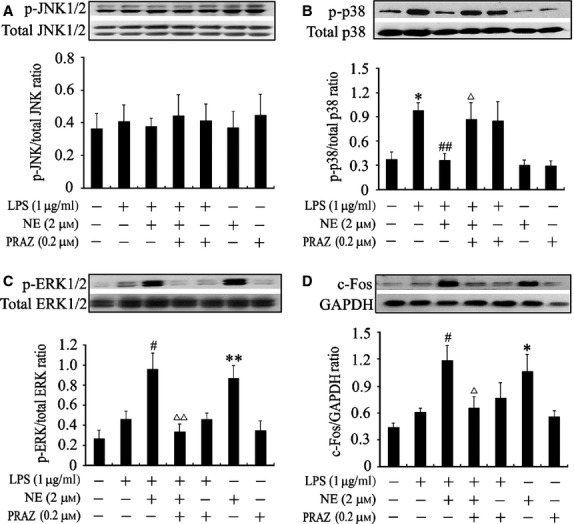
Effects of norepinephrine (NE) and prazosin (PRAZ) on lipopolysaccharide (LPS)-induced JNK1/2, p38 and extracellular signal-regulated kinase 1/2 (ERK1/2) phosphorylation and c-Fos expression in neonatal rat cardiomyocytes. After pre-treatment with PRAZ or vehicle for 30 min., cardiomyocytes were incubated with NE or vehicle for 10 min. and then with LPS or normal saline for another 30 min. Representative blots and quantification of JNK1/2 (A), p38 (B) and ERK1/2 (C) phosphorylation and c-Fos (D) expression are shown. Data are expressed as mean ± SEM, *n* = 5. *P < 0.05, **P < 0.01 *versus* control group, ^#^*P* < 0.05, ^##^*P* < 0.01 *versus* LPS group, ^Δ^*P* < 0.05, ^ΔΔ^*P* < 0.01 *versus* LPS+NE group.

It is well recognized that MAPK and NF-κB activation as well as c-Fos expression involve the regulation of LPS-induced TNF-α expression in cardiomyocytes 2. Thus, we investigated the effects of NE on p38 MAPK, ERK1/2 and JNK1/2 phosphorylation, NF-κB activation as well as c-Fos expression in LPS-stimulated cardiomyocytes in the presence or absence of prazosin. Cardiomyocytes were pre-treated with prazosin (0.2 μM) or vehicle for 30 min., followed by NE (2 μM) incubation for 10 min., and then stimulated with LPS for another 30 min. JNK1/2, p38 and ERK1/2 phosphorylation, c-Fos expression as well as NF-κB translocation were examined by Western blotting and immunofluorescence analysis respectively. Treatment with prazosin, NE or/and LPS had no marked effects on JNK1/2 phosphorylation (Fig. 2A). However, LPS significantly increased the p38 phosphorylation by 165% in cardiomyocytes compared with control (*P* < 0.05), NE-pre-treated cardiomyocytes in the presence of LPS showed a marked decrease (63%) in p38 phosphorylation compared with cells stimulated with LPS only (*P* < 0.01), this action of NE was almost completely reversed by prazosin, while prazosin did not affect the phosphorylation of p38 in LPS-challenged cardiomyocytes (Fig. 2B). These data indicates that NE inhibits LPS-induced p38 phosphorylation *via* α_1_-AR in cardiomyocytes.

As shown in Figure 2C and D, LPS at 1 μg/ml failed to significantly elevate ERK1/2 phosphorylation and c-Fos expression compared with control, whereas NE markedly increased the phosphorylation of ERK1/2 and c-Fos expression by 109% and 95%, respectively, in LPS-stimulated cardiomyocytes, which was prevented by prazosin. In contrast, prazosin did not alter ERK1/2 phosphorylation and c-Fos expression in LPS-stimulated cardiomyocytes. In addition, NE alone induced an increase in the phosphorylation of ERK1/2 and c-Fos expression in cardiomyocytes (*P* < 0.01, *P* < 0.05). These results demonstrate that NE potentiates ERK1/2 phosphorylation and c-Fos expression *via* α_1_-AR in LPS-treated cardiomyocytes.

**Fig 3:**
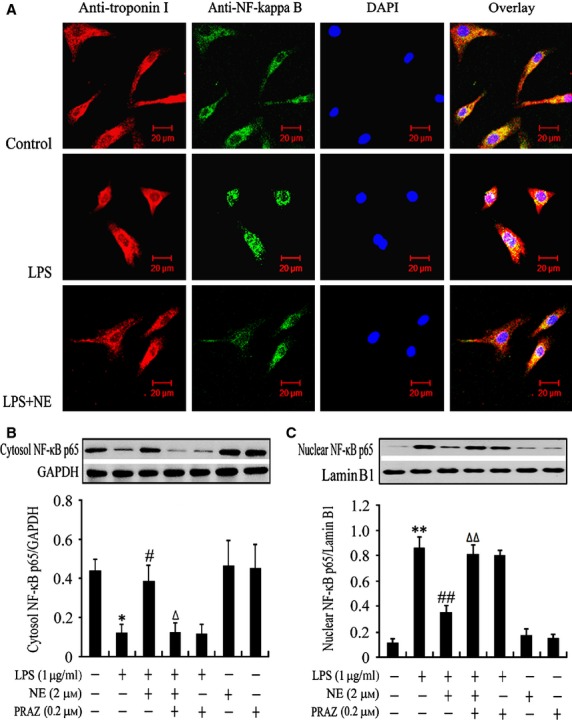
Effects of norepinephrine (NE) and prazosin (PRAZ) on lipopolysaccharide (LPS)-induced NF-κB activation in neonatal rat cardiomyocytes. Cardiomyocytes were treated as described in Figure 2. (A) NF-κB p65 nuclear translocation was analysed by laser confocal microscopy. Scale bar = 20 μm. (B and C) The cytosolic and nuclear NF-κB p65 levels were assessed by western blot; data are expressed as mean ± SEM, *n* = 5. *P < 0.05, **P < 0.01 *versus* control, ^#^*P* < 0.05, ^##^*P* < 0.01 *versus* LPS group, ^Δ^*P* < 0.05, ^ΔΔ^*P* < 0.01 *versus* LPS+NE group.

As we expected, LPS stimulation for 30 min. caused an increase in nuclear translocation of NF-κB p65, which was prevented by NE pre-treatment (Fig. 3A). Furthermore, LPS also significantly reduced cytosolic NF-κB p65 levels by 72% and increased nuclear NF-κB p65 levels by 616% in cardiomyocytes compared with control (*P* < 0.05), which was prevented by NE pre-treatment (*P* < 0.05). In contrast, prazosin administration abolished the effects of NE on cytosolic and nuclear NF-κB p65 levels in LPS-challenged cardiomyocytes (*P* < 0.05). However, prazosin did not affect the cytosolic and nuclear NF-κB p65 levels in cardiomyocytes stimulated with or without LPS (Fig. 3B and C). These findings suggest that NE suppresses LPS-induced NF-κB activation through binding to α_1_-AR in cardiomyocytes.

### U0126 reverses the effect of NE on c-Fos expression, p38 phosphorylation and TNF-α production, but not on NF-κB activation in LPS-challenged cardiomyocytes

**Fig 4:**
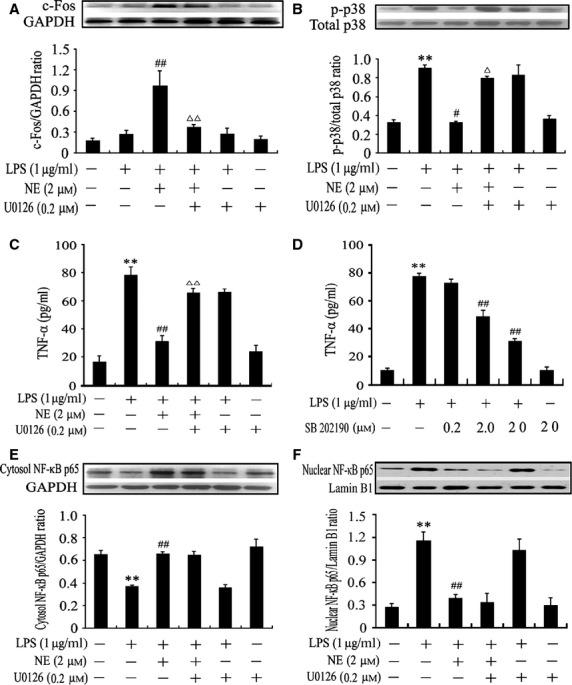
Norepinephrine (NE) enhances c-Fos expression, inhibits p38 mitogen-activated protein kinase and in turn partly decreased tumour necrosis factor α (TNF-α) production, but not NF-κB activation, *via* activating extracellular signal-regulated kinase 1/2 (ERK1/2) signal pathway in lipopolysaccharide (LPS)-challenged cardiomyocytes. After pre-treatment with ERK1/2 inhibitor (U0126), p38 inhibitor (SB 202190) or vehicle for 30 min., cardiomyocytes were stimulated with NE or vehicle for 10 min. and then exposed to LPS or normal saline for additional 30 min. (A, B, E and F) or 6 hrs (C and D). Expression of c-Fos (A), p38 phosphorylation (B), cytosolic (E) and nuclear (F) NF-κB p65 levels were determined by western blot. TNF-α level in the supernatant was detected by ELISA (C and D). Data are mean ± SEM, *n* = 5–6. **P < 0.01 *versus* control, ^#^*P* < 0.05, ^##^*P* < 0.01 *versus* LPS group, ^Δ^*P* < 0.05, ^ΔΔ^*P* < 0.01 *versus* LPS+NE group.

The previous studies demonstrated that inhibition of ERK 1/2 abolished the NE-induced increase in c-Fos expression in cardiomyocytes 23 and c-Fos inhibits p38 signalling, resulting in decreased TNF-α response to LPS in cardiomyocytes 24. To demonstrate the role of ERK1/2 in the effect of NE on c-Fos expression, p38 phosphorylation, NF-κB activation and TNF-α production in LPS-challenged cardiomyocytes, we used U0126 to inhibit ERK1/2 signalling pathway. As shown in Figure 4A–C, NE promoted c-Fos expression and reduced the phosphorylation of p38 in LPS-treated cardiomyocytes; it also suppressed LPS-induced TNF-α production in cardiomyocytes at 6 hrs after LPS exposure. U0126 pre-treatment increased p38 phosphorylation by 147%, decreased c-Fos expression by 62% in response to NE and partly reversed the inhibitory effect of NE on TNF-α production (*P* < 0.01) in LPS-stimulated cardiomyocytes. Exposure of control or LPS-treated cardiomyocytes to U0126 had no effect on c-Fos expression, p38 phosphorylation and TNF-α production. In addition, pre-treatment with SB202190, a p38 MAPK inhibitor, significantly suppressed LPS-induced TNF-α production in cardiomyocytes in a dose-dependent manner (Fig. 4D). On the other hand, cytosolic NF-κB p65 level was significantly decreased (*P* < 0.01) and nuclear NF-κB p65 level was significantly elevated (*P* < 0.01) in LPS-stimulated cardiomyocytes, which was markedly reversed by NE (*P* < 0.01), while U0126 did not abolish the effects of NE on cytosolic and nuclear NF-κB p65 levels in LPS-stimulated cardiomyocytes (Fig. 4E and F). These findings suggest that ERK1/2 – c-Fos signalling activation induced by NE inhibits p38 MAPK, but not NF-κB activation, and in turn partly suppresses TNF-α production in LPS-challenged cardiomyocytes.

### Phenylephrine mimics the effect of NE on LPS-challenged cardiomyocytes and attenuates cardiac dysfunction in endotoxaemic mice

**Fig 5:**
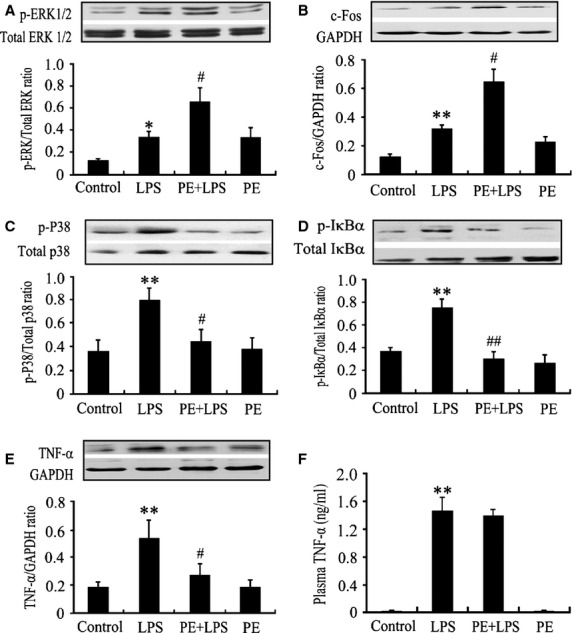
Effects of α_1_-AR agonists, phenylephrine (PE), on lipopolysaccharide (LPS)-induced myocardial extracellular signal-regulated kinase 1/2 (ERK1/2), p38 and IκBα phosphorylation, c-Fos expression as well as myocardial and plasma tumour necrosis factor α (TNF-α) production in mice. BALB/c mice were challenged with LPS (20 mg/kg), and PE (20 μg/kg) was injected subcutaneously 30 min. before and 2 hrs after LPS administration respectively. At 2.5 hrs after LPS administration, myocardial ERK1/2 (A), p38 (C) and IκB (D) phosphorylation, c-Fos expression (B), myocardial (E) and plasma (F) TNF-α levels were examined by western blot or ELISA. Data are mean ± SEM, *n* = 8. *P < 0.05, **P < 0.01 *versus* control, ^#^*P* < 0.05, ^##^*P* < 0.01 *versus* LPS group.

**Fig 6:**
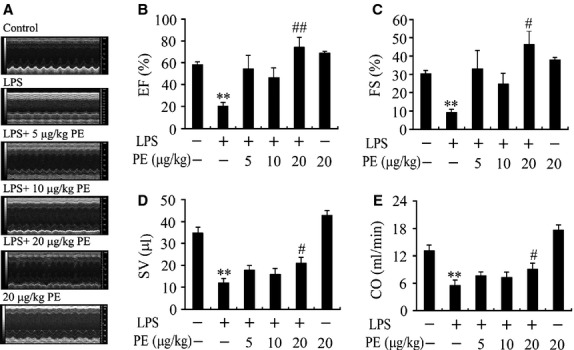
Effect of phenylephrine (PE) on cardiac function in endotoxaemic mice. Mice were challenged with LPS (20 mg/kg), and PE (5, 10 or 20 μg/kg) was injected subcutaneously 30 min. before and 2 hrs after LPS administration respectively. (A) The representative M-mode echocardiograms at 12 hrs after LPS administration. (B) LV ejection fraction (EF), (C) fractional shortening (FS), (D) stroke volume (SV) and (E) cardiac output (CO) are presented. Data are mean ± SEM, *n* = 7–10. **P < 0.01 *versus* control, ^#^*P* < 0.05, ^##^*P* < 0.01 *versus* LPS group.

To determine whether α_1_-AR activation suppresses myocardial TNF-α production *via* modulating ERK1/2 – c-Fos – p38 and NF-κB signalling pathway *in vivo*, PE, an α_1_-AR agonist, was used in a murine model of endotoxaemia. As depicted in Figure 5, LPS markedly increased TNF-α and c-Fos expression as well as ERK1/2, p38 and IκBα phosphorylation in the myocardium compared with sham group (*P* < 0.05, *P* < 0.01). Treatment with PE (20 μg/kg) further enhanced ERK1/2 phosphorylation and c-Fos expression (94% and 103% respectively), while inhibited TNF-α production by 50% as well as p38 and IκBα phosphorylation (44% and 60% respectively) in the myocardium of endotoxaemic mice. In contrast, PE did not decrease plasma TNF-α level in endotoxaemic mice. PE alone did not significantly affect myocardial TNF-α and c-Fos expression as well as ERK1/2, p38 and IκBα phosphorylation. Moreover, LPS caused significant decreases in EF, FS, SV and CO, which were prevented by 20 μg/kg PE treatment. Treatment with PE alone did not affect cardiac function in control mice; there was no significant difference in EF, FS, SV and CO (all *P* > 0.05) between PE and control groups (Fig. 6).

## Discussion

It is well-established that cardiomyocyte TNF-α production contributes to cardiac dysfunction in sepsis 2–6 and circulating NE levels elevate significantly during sepsis 13–16. Therefore, it is very important to investigate the effect of NE on LPS-induced cardiomyocyte TNF-α production and the underlying mechanisms to improve the existing and rather ineffective therapy for septic cardiomyopathy.

A previous study demonstrated that circulating NE level could reach 20 nM during sepsis 16. Importantly, NE has been regarded as a first-line agent for the treatment of septic shock 20. Thus, we examined the effects of 2–2000 nM NE on LPS-induced cardiomyocyte TNF-α production in this study. The results demonstrated for the first time to our knowledge that NE significantly suppressed LPS-stimulated TNF-α production in a concentration-dependent manner in cardiomyocytes. To identify which AR subtype is involved in the action of NE, we used α_1_-AR antagonist prazosin, β_1_-AR antagonist atenolol and β_2_-AR antagonist ICI 118,551 in the next experiments and found that only prazosin pre-treatment abolished the inhibitory effect of NE on TNF-α production and mRNA expression in LPS-challenged cardiomyocytes. Particularly, an α_1_-AR agonist, PE, also inhibited TNF-α production in a dose-dependent manner in LPS-treated cardiomyocytes. These results suggest that α_1_-AR is responsible for NE-induced suppression of TNF-α expression in LPS-treated cardiomyocytes. Interestingly, we observed that both β_1_-and β_2_-AR antagonists prevented LPS-induced TNF-α secretion in cardiomyocytes. Huang *et al*. found that endogenous NE constitutively produced by intrinsic cardiac adrenergic cells affected the spontaneous beating rate of neonatal rat cardiomyocytes *via* β-AR *in vitro* 25. We preliminarily observed that β_1_-AR agonist enhanced LPS-induced TNF-α secretion in cardiomyocytes (data not shown). Hence, it is possible that β_1_-or β_2_-AR antagonist may inhibit LPS-induced TNF-α secretion in neonatal rat cardiomyocytes by abolishing action of catecholamine released from intrinsic cardiac adrenergic cells *via* its β-AR inhibitory activities; this remains to be further investigated.

Accumulating evidence indicates that activation of MAPK signal pathways represents an important mechanism leading to increased cardiomyocyte TNF-α production caused by LPS 2. Lipopolysaccharide induced p38 phosphorylation and TNF-α expression in cardiomyocytes, selective inhibition of p38 abrogated LPS-induced cardiomyocyte TNF-α expression 26,27. Similarly, LPS also rapidly increased ERK1/2 phosphorylation in neonatal mouse cardiomyocytes, and inhibition of ERK1/2 abolished LPS-induced TNF-α production in cardiomyocytes 27–29. In contrast, JNK1 deficiency promoted LPS-stimulated cardiomyocyte TNF-α expression 24. In this study, we observed that treatment with 1 μg/ml LPS for 30 min. significantly induced p38 phosphorylation in cardiomyocytes. Norepinephrine markedly inhibited LPS-induced p38 phosphorylation, which was almost completely reversed by prazosin pre-treatment. These data indicate that α_1_-AR activation by NE reduced LPS-induced p38 activation in neonatal rat cardiomyocytes. However, NE that activates α_1_-AR did not induce p38 phosphorylation in normal rat cardiomyocytes (Fig. 2B) and we did not observe any change in myocardial p38 phosphorylation after PE treatment in normal control mice (Fig. 5C). These results are inconsistent with an earlier report that PE treatment caused p38 phosphorylation in isolated adult rat ventricular myocytes, suggesting that stimulation of α_1_-AR leads to cardiomyocyte p38 activation 30. In this study, rat cardiomyocyte and mouse myocardial p38 phosphorylation were detected at 40 min. after treatment with 2 μM NE and 30 min. after the second subcutaneous injection of PE, respectively, whereas p38 phosphorylation was examined in rat cardiomyocytes at 10 min. after stimulation with 5 μM PE in the previous study 30. It has been demonstrated that treatment with PE for 10 min. induced cardiomyocyte p38 phosphorylation *via* protein kinase C (PKC)_δ_ and PKC_ε_ activation 30 and the activation of PKC_δ_ and PKC_ε_ peaked within 1 min. and slowly returned towards basal level within 15 min. after PE treatment 31, another study also showed that cardiomyocyte p38 phosphorylation increased markedly 5 min. after PE treatment and that phosphorylation declined after 15 min. towards baseline levels 32. Thus, the above inconsistency on p38 activation may be largely as a result of the different time-point of p38 phosphorylation determination. In addition, we observed that incubation with 1 μg/ml LPS failed to significantly cause JNK1/2 and ERK1/2 phosphorylation in neonatal rat cardiomyocytes. However, the other studies demonstrated that LPS treatment rapidly increased ERK1/2 and JNK1/2 phosphorylation in cardiomyocytes 28,29. Although it is difficult to explain this inconsistency, it is reasonable to speculate that some factors, such as LPS concentration and species, may contribute to these discrepant results. In the previous study 28,29, the ERK1/2 and JNK1/2 phosphorylation were determined in neonatal mouse cardiomyocytes exposed to 10 μg/ml LPS, whereas neonatal rat cardiomyocytes were stimulated with 1 μg/ml LPS in this study. Future study is required to clarify this issue. Interestingly, our data showed that NE dramatically increased ERK1/2 phosphorylation and c-Fos expression in LPS-challenged cardiomyocytes, which were prevented by prazosin. These findings suggest that NE enhanced ERK1/2 phosphorylation and c-Fos expression *via* activating α_1_-AR in LPS-challenged cardiomyocytes. In support of these observations, other studies have also demonstrated that NE can activate ERK1/2 and in turn increase c-Fos expression *via* stimulating α_1_-AR in normal adult rat cardiomyocytes 23,33. Recently, Peng *et al*. showed that c-Fos overexpression reduced LPS-induced TNF-α expression in cardiomyocytes, which was associated with a reduction in p38 phosphorylation 24. Accordingly, we hypothesized that NE may increase c-Fos expression, in turn inhibit p38 phosphorylation and TNF-α production *via* activating ERK1/2 signalling pathway in LPS-challenged cardiomyocytes. To test this hypothesis, we further examined the effect of ERK1/2 inhibitor, U0126, on c-Fos expression, p38 phosphorylation and TNF-α production in NE or/and LPS-treated cardiomyocytes. As LPS stimulation for 30 min. can result in ERK1/2 and p38 phosphorylation in neonatal rat cardiomyocytes and transient elevation of c-Fos protein within 1 hr after stimulation was found in neonatal rat cardiomyocytes 24,34, cardiomyocyte c-Fos expression and p38 phosphorylation were examined 30 min. after LPS stimulation in this study. We found that NE enhanced c-Fos expression and decreased p38 phosphorylation in LPS-treated cardiomyocytes, which were reversed by U0126 pre-treatment. Moreover, U0126 largely reversed the inhibitory effects of NE on LPS-induced TNF-α production in cardiomyocytes, and pre-treatment with SB202190, a p38 MAPK inhibitor, also inhibited LPS-induced TNF-α production in a dose-dependent manner in cardiomyocytes. Taken together, our data suggest that NE stimulates ERK phosphorylation and c-Fos expression, leading to decreased p38 activation and TNF-α expression *via* activating α_1_-AR in LPS-treated cardiomyocytes, and p38 activation is a major event in LPS-induced cardiomyocyte TNF-α expression.

On the other hand, NF-κB activation has also been shown to mediate LPS-induced TNF-α expression in cardiomyocytes 35. Wright *et al*. demonstrated that LPS-induced TNF-α production *via* activating NF-κB pathway in cultured neonatal cardiomyocytes, demonstrated by the degradation of IκB, the appearance of NF-κB-binding complexes in cardiomyocyte nuclear extracts and the inhibition of LPS-stimulated TNF-α expression by inhibitors of NF-κB activation 36. We also found that LPS significantly induced NF-κB activation in cardiomyocytes; increased NF-κB p65 nuclear translocation, elevated nuclear NF-κB p65 level and reduced cytosolic NF-κB p65 level were observed at 30 min. after LPS stimulation in cardiomyocytes. In addition, NE pre-treatment suppressed NF-κB activation in LPS-challenged cardiomyocytes, and this NE effect was abrogated by prazosin, but not U0126 pre-treatment. These observations indicate that NE inhibits LPS-induced NF-κB activation in cardiomyocytes *via* stimulating α_1_-AR, which is independent of ERK1/2 signalling pathway. However, it remains unclear how NE inhibits NF-κB activation through α_1_-AR in LPS-challenged cardiomyocytes. It has been well known that activation of calcium and PKC signal pathways are important downstream events for α_1_-AR stimulation 37. Turrell *et al*. demonstrated that PE activated PKC_ε_ and PKC_δ_ leading to p38 activation in cardiomyocytes, which induced an increase in the peak sarcolemmal ATP-sensitive K^+^ current and a subsequent decrease in Ca^2+^ loading during stimulation 30. Rao *et al*. observed that PE increased ERK1/2 activity in cardiomyocytes *via* a pathway dependent on PKC_ε_ 32. Importantly, some studies have shown that intracellular Ca^2+^ levels are elevated by LPS, which contribute to TNF-α expression in cardiomyocytes 29,38; other studies demonstrated that PKC plays a regulatory role in cardiomyocyte TNF-α secretion. For example, burn serum activated PKC_α_, PKC_δ_ and PKC_ε_ in cardiomyocytes and caused TNF-α expression, inhibition of PKC_ε_ prevented burn serum-related cardiomyocyte TNF-α secretion 39. Receptor activator of NF-κB ligand increased TNF-α production in cardiomyocytes, which involves PKC-NF-κB-mediated mechanisms 40. Accordingly, it is likely that calcium and PKC signal pathways may involve the suppression of NF-κB activation and TNF-α production by α_1_-AR activation in LPS-challenged cardiomyocytes; this needs to be further investigated.

To confirm the current observations, we further examined the effect of PE, a selective α_1_-AR agonist, on the phosphorylation of ERK1/2, p38 and IκBα, expression of c-Fos and TNF-α in the myocardium as well as cardiac dysfunction in a mouse model of endotoxaemia. The results demonstrated that PE attenuated cardiac dysfunction in endotoxaemic mice, as demonstrated by improved EF, FS, SV and CO. Meanwhile, PE not only enhanced ERK1/2 phosphorylation and c-Fos expression but also inhibited p38 and IκBα phosphorylation and reduced TNF-α expression in the myocardium of endotoxaemic mice. However, PE did not affect circulatory TNF-α level in endotoxaemic mice. Although *in vivo* effects of ERK activation on myocardial TNF-α production in endotoxaemia need to be investigated, some studies have shown that inhibition of p38 activation or cardiomyocyte NF-κB activation is sufficient to reduce cardiac TNF-α expression and prevent cardiac dysfunction in endotoxaemia 41,42. Therefore, it seems reasonable to speculate that cardiomyocyte α_1_-AR activation may inhibit myocardial TNF-α production and prevent cardiac dysfunction *via* reducing myocardial NF-κB and p38 activation in endotoxaemic mice, and decreased myocardial p38 activation by α_1_-AR stimulation may be associated with ERK/c-Fos signalling activation during endotoxaemia.

In conclusion, our results demonstrate that NE inhibits LPS-induced TNF-α expression in cardiomyocytes *via* suppressing NF-κB and p38 signalling pathways in an α_1_-AR-dependent manner, and stimulation of α_1_-AR reduces LPS-triggered p38 phosphorylation by activating ERK-c-Fos signalling pathway in cardiomyocytes. Furthermore, activation of the α_1_-AR can decrease myocardial TNF-α expression, maybe *via* activating ERK-c-Fos signalling and inhibiting NF-κB signalling, and improve cardiac dysfunction in endotoxaemia. These findings define specific signalling molecular events that mediate the inhibitory effect of NE on LPS-induced TNF-α production in cardiomyocytes, and may provide potentially valuable therapeutic targets for the treatment of myocardial depression during sepsis.
